# Supplementary material for: The History of Infectious Diseases and Medicine
Source: Pathogens. 2022 Oct 4;11(10):1147. doi: 10.3390/pathogens11101147 (PMC9609744; doi:10.3390/pathogens11101147)
Supplement: Supplementary file 1 [file pathogens-11-01147-s001.zip › Suppl_MedicalHistory.pdf]

The medical publications and textbooks in the five historical periods

1. Classic Western medicine (Ancient times to the 15th century)

1.1. Hippocratic Corpus

Around seventy documents associated with him and his students were assembled to form the so-called Hippocratic Corpus (Latin: *Corpus Hippocraticum*), including the *Epidemiai* (ἐπιδημίαι) volumes I-VII. The title word "*Epidemiai*" does not indicate our current understanding of the word "epidemic," but merely indicates visits to a distant place or person, related to seasonal outbreaks of disease, or the lengthy sojourns made by itinerant doctors[1]. Some terms in the Hippocratic Corpus may appear to represent infectious diseases in the modern sense, but a careful reading would show that their meanings are actually equivocal. For example, the word 'plague' (λοιμός) represented diseases with high mortality in general, 'cholera' (χόλερα) the symptoms of diarrhea and vomiting at the same time, and 'dysentery' (δυσεντερία) the symptoms of frequent watery stools containing blood and mucus. This was long before the discovery of infectious pathogens, and the concept of infectious diseases had yet to be invented.

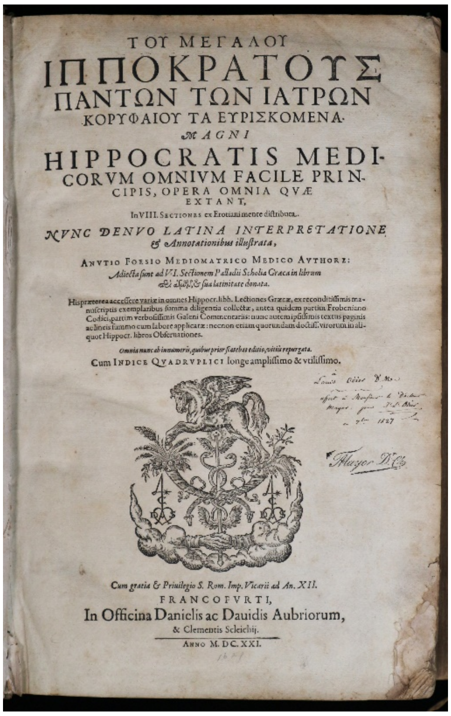

Upright figure.

"Hippocratic corpus" (1621). Personal collection of Tatsuo Sakai.

1.2. Galen's treatises

Galen of Pergamon wrote numerous treatises on various aspects of medicine including general medicine, natural sciences (physics), anatomy, physiology, dietetics, pathology, semeiotics, and pharmaceutics. His treatises were widely adopted and regarded as authoritative into the Middle Ages and the Renaissance, earning him the title of "the Prince of Medicine."[2] He witnessed the Antonine Plague during his stay in Rome. The described the symptoms in his *Method of Medicine*, V.12, which is today which is today considered smallpox[3].

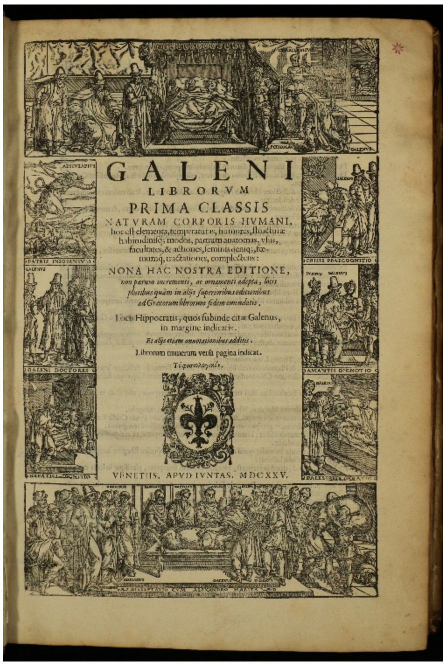

Upright figure.

Galen "Opera omnia" (1625). Personal collection of Tatsuo Sakai.

1.3. Classic medical textbooks in the middle ages

After the division and decline of the Roman Empire in 395, the Galenic treatises were translated into Syriac and Arabic and transmitted to the Orient, other medical works such as Avicenna's encyclopedic *Liber Canonis (Canon of Medicine)* were compiled by the synthetic exposition of classical Greek medicine.

At the Schola Medica Salernitana beginning from the later 10th century compiled a teaching material *Articella* incorporating Hippocratic, Galenic and other documents translated from Arabic and Greek texts.

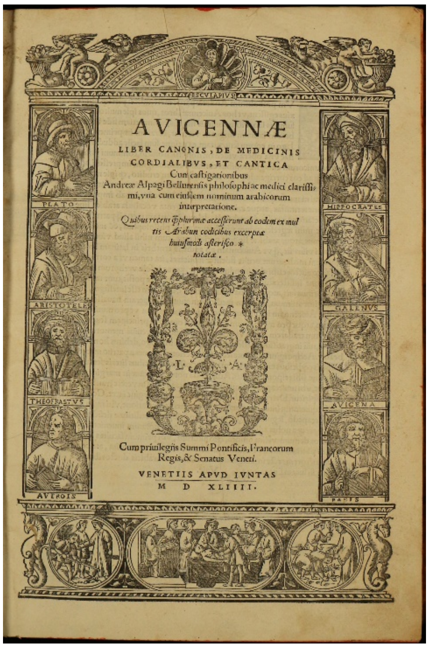

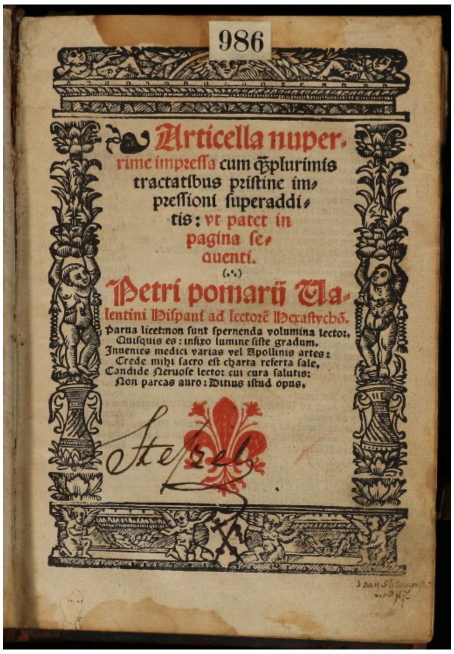

Upright figure.

Avicenna "Liber canonis" (1544). Personal collection of Tatsuo Sakai.

Articella (1534). Personal collection of Tatsuo Sakai.

2. Traditional Western medicine in the 16th to 18th centuries

2.1. Vesalius "Fabrica" and following anatomical studies

Andreas Vesalius's *De humani corporis fabrica* (On the structure of the human body, 1543) is the outstanding medical literature in this period. His seven-volume work reexamined the description of Galenic anatomy with human dissection and visualized the human body structures with numerous precise anatomical drawings. The publication of *Fabrica* was revolutionary in that it introduced a shift away from the hitherto target of medical studies to better understand the classical medical texts to the new scientific investigation of phenomena in nature including the human body.

Thereafter anatomical research brought about numerous discoveries, notably new findings on blood circulation by William Harvey (*Exercitatio anatomica de motu cordis et sanguinis in animalibus*, 1628), on the lymphatic system by Thomas Bartholin (*Vasa lymphatica,* 1653) and on the brain structure by Thomas Willis (*Cerebri anatome*, 1664)[4].

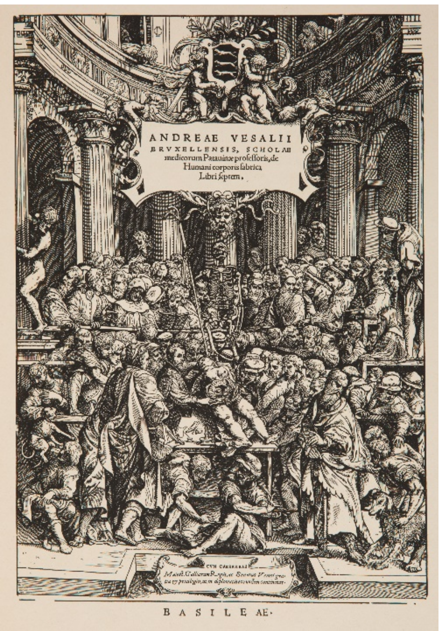

Upright figure.

Title page of Vesalius "Fabrica" (1543), from "Icones Anatomicae" (1934). Personal collection of Tatsuo Sakai.

2.2. Infectious diseases in the *Practica* books

Daniel Sennert's *De febribus libri IV* (1619)[5]. In *De febribus libri IV*, a malaria-like intermittent fever was described in book two "Of the putrid fevers", and small pox and measles in book four "Of the pestilence, and the pestilential and malignant fevers".

Daniel Sennert's *Practicae medicinae* in 6 vols. (1628-1635)[6]. book three, part two described dysentery as an abdominal illness, and book six, part three was devoted to the topic "Of occult diseases from water, air, and contagions, and of contagious diseases in general" and part four to that "Of the venereal pox".

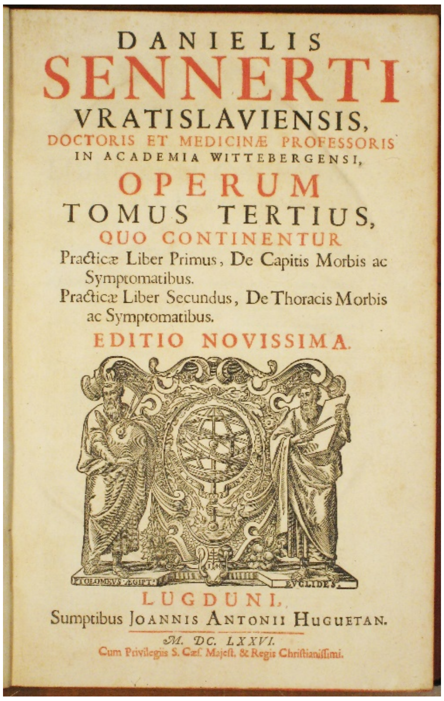

Upright figure.

Sennert D "Operum" (1676) including *Practica*. Personal collection of Tatsuo Sakai.

Table S1. Sennert J "De febribus libri IV" (1619), "Practicae medicinae" 6 vols. (1628-1635)

"De febribus libri IV" (1619)	
Book 1	Of the fevers in general and ephemeral fevers
Book 2	Of the putrid fevers
Book 3	Of the hectic fevers
Book 4	Of the pestilence, and the pestilential and malignant fevers.

"Practicae medicinae" 6 vols. (1628-1635)	
Book 1 (1628)	
Part 1	On the diseases of the head
Part 2	On the symptoms that occur in the internal senses and in the brain
Part 3	
Section 1	On the injured touch.
Section 2	On the disease and symptoms of the eye
Section 3	On the disease and symptoms of the ears
Section 4	On the disease and symptoms of the nose
Section 5	On the disease and symptoms of the tongue
Book 2 (1629)	
Part 1	On the mouth and throat, and the parts contained in these fields.
Part 2	On the unnatural affections of the trachea, lungs, mediastinum, diaphragm, and thorax.
Part 3	On the symptoms that affect the lungs and the chest.
Part 4	On the diseases and symptoms of the heart.

Table S1. Continued

Book 3 (1631)	
Part 1	
Section 1	On the diseases of the esophagus and the stomach
Section 2	On the symptoms of the stomach
Part 2	
Section 1	On the diseases of the intestines
Section 2	On the symptoms that affect the intestines.
Part 3	On the diseases of the mesentery, pancreas and omentum
Part 4	On the unnatural affections of the spleen
Part 5	
Section 1	On the hypochondriac affections.
Section 2	On the scurvy
Part 6	
Section 1	On the diseases of the liver
Section 2	On the symptoms that occur to the liver
Part 7	
Section 1	On the diseases of the kidneys and ureters.
Section 2	On the symptoms of the kidneys.
Part 8	
Section 1	On the diseases of the bladder
Section 2	On the symptoms of the bladder
Part 9	
Section 1	On the diseases of the genital parts in men
Section 2	On the symptoms that occur in the genital organs and during generation in men
Part 10	On the unnatural affections of the navel and abdomen
Book 4 (1632)	
Part 1	
Section 1	Of the diseases of the pudenda and the cervix of uterus in women
Section 2	Of the diseases of the uterus itself
Part 2	
Section 1	Of the symptoms that occur in the uterus in women.
Section 2	On the symptoms that occur during menstruation, and other unnatural discharges from the uterus.
Section 3	On the symptoms, that befall almost all virgins and women after puberty in the uterus.
Section 4	On the symptoms that occur during pregnancy
Section 5	On the government of the pregnant women, and the unnatural affections which affect the pregnant women.
Section 6	On the symptoms that occur at birth.
Section 7	On the government of puerperants, and the unnatural affections which occur to them after childbirth.
Part 3	On the unnatural affections of the breasts
Section 1	On the diseases of the breasts
Section 2	On the symptoms of the breasts
Treatise on the diseases of infants	
Part 1	Of the diet and government of infants
Part 2	Of diseases and symptoms of children
Book 5 (1634)	
Part 1	Of tumors
Part 2	Of ulcers
Part 3	Of the vices of the skin, hair, and nails
Section 1	Of the vices of the skin
Section 2	Of things amiss in the hair and nails
Part 4	Of wounds
Part 5	Of fractures
Part 6	Of luxations

Table S1. Continued

Book 6 (1635)	
Part 1	Of diseases from occult qualities in general
Part 2	Of occult, malignant, and venemous diseases arising from the internal fault of the humors
Part 3	Of occult diseases from water, air, and contagions, and of contagious diseases in general
Part 4	Of the venereal pox
Part 5	Of outward poisons in general
Part 6	Of poisons from minerals and metals
Part 7	Of poisons from plants
Part 8	Of poisons that come from animals
Part 9	Of diseases by witchcraft, incantation, and charms

A supplemental document describing the detail of each section is available at the following link.

https://doi.org/10.6084/m9.figshare.19246044.v1

3. Early Modern Medicine (19th century)

Niemeyer's *Lehrbuch der speciellen Pathologie und Therapie* (1858) [7] was the most popular textbook of medicine in the middle third of the 19th century. It dealt mainly with the diseases of various organs, and recognized various acute and chronic infectious diseases in the section of "constitutional diseases" (volume 2, part 2, section 3).

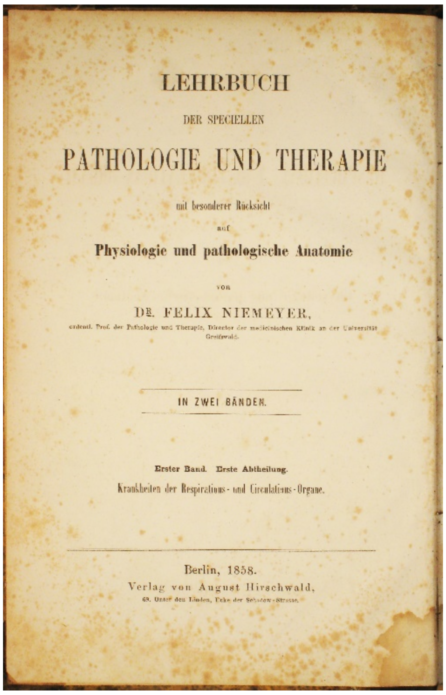

LEHRBUCH

DER SPECIELLEN

PATHOLOGIE UND THERAPIE

mit besonderer Rücksicht

auf

Physiologie und pathologische Anatomie

von

DR. FELIX NIEMEYER,

ordentl. Prof. der Pathologie und Therapie, Director der medicinischen Klinik an der Universität Greifswald.

IN ZWEI BÄNDEN.

Erster Band. Erste Abtheilung.

Krankheiten der Respirations- und Circulations-Organe.

Berlin, 1858.

Verlag von August Hirschwald,

68. Unter den Linden, Ecke der Schadow-Strasse.

Upright figure.

Niemeyer F "Lehrbuch der speciellen Pathologie und Therapie" (1858).

Personal collection of Tatsuo Sakai

Table S2. Niemeyer F "Lehrbuch der speciellen Pathologie und Therapie" (1858-61)

Volume 1 (1858)

Part 1	The diseases of the respiratory and circulatory organs with special reference to physiology and pathological anatomy
Diseases of the respiratory organs	
Section 1	Diseases of the larynx
Section 2	Diseases of the trachea and bronchi
Section 3	Diseases of the pulmonary parenchyme
Section 4	Diseases of the pleura
Diseases of the circulatory organs	
Section 1	Diseases of the heart
Section 2	Diseases of the pericardium
Section 3	Diseases of the great vessels

Table S2. Continued

Part 2	The diseases of the digestive organs, the liver and the spleen with special refernce to physiology and pathological anatomy
Diseases of the digestive organs	
Section 1	Diseases of the oral cavity
Section 2	Diseases of the pharynx
Section 3	Diseases of the exophagus
Section 4	Diseases of the stomach
Section 5	Diseases of the intestinal canals
Section 6	Diseases of the peritoneum
Diseases of the liver and bile ducts	
Section 1	Diseases of the liver
Section 2	Diseases of the bile ducts
Section 3	Diseases of the spleen

Volume 2 (1861)

Part 1	The diseases of the urinary and genital organs, the nerve centers and the nerves with special reference to physiology and pathological anatomy
Diseases of the urinary organs	
Section 1	Diseases of the kidney
Section 2	Diseases of the renal pelvis and ureter
Section 3	Diseases of the urinary bladder
Section 4	Diseases of the urethra
Diseases of the genital organs	
A	Diseases of the male genital organs
B	Diseases of the female genital organs
Section 1	Diseases of the ovary
Section 2	Diseases of the uterus
Section 3	Diseases of the vagina
Diseases of the nervous system	
Section 1	Diseases of the brain
Section 2	Diseases of the spinal cord and its coat
Section 3	Diseases of the peripheral nerves
Section 4	Expanded neurosis with unknown anatomical basis
Part 2	The diseases of the skin, the locomotor organs and the constitutional diseases with special reference to physiology and pathological anatomy
Diseases of the skin	
Diseases of the locomotor organs	
Constitutional diseases	
Section 1	Acute infectious diseases
Section 2	Chronic infectious diseases
Section 3	General nutritional abnormalities that do not depend on an infection

A supplemental document describing the detail of each section is available at the following link.

https://doi.org/10.6084/m9.figshare.19246047.v2

4. Late Modern Medicine (20th century)

Osler, William (1849-1919) was the most prominent physician in the early 20th century, and his *Principles and Practice of Medicine* (1st ed. 1892, 9th edition, 1920)[9] was the best English work on medicine of its time. The infectious diseases were allotted to a separate and the first section of the textbook.

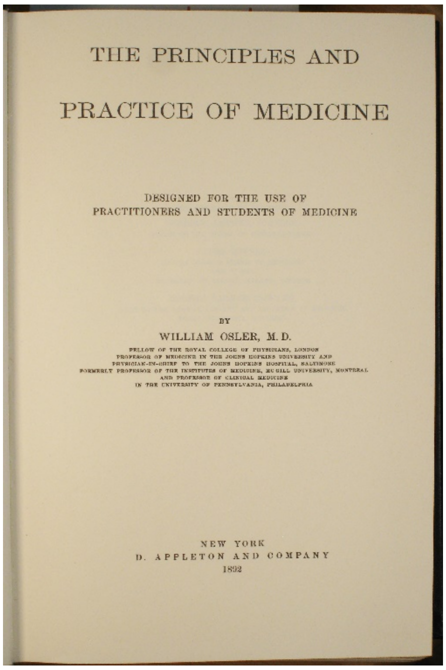

Upright figure.

Osler W "Principles and Practice of Medicine" (1892). Personal collection of Tatsuo Sakai

Table S3. Osler W "The principles and practice of medicine" (1892)

Section 1	Specific infectious diseases.
Section 2	Constitutional diseases.
Section 3	Diseases of the digestive system.
Section 4	Diseases of the respiratory system.
Section 5	Diseases of the circulatory system.
Section 6	Diseases of the blood and ductless glands.
Section 7	Diseases of the kidneys.
Section 8	Diseases of the nervous system.
Section 9	Diseases of the muscles.
Section 10	The intoxications; Sun-stroke; Obesity.
Section 11	Diseases due to animal parasites.

A supplemental document describing the detail of each section is available at the following link.

https://doi.org/10.6084/m9.figshare.19246050.v2

5. Exact Medicine (1990 - to date)

Harrison's Principles of Internal Medicine, 20th edition (2018) [10]

Table S4. Harrison's Principles of Internal Medicine, 20e (2018)

Part 1	The Profession of Medicine
Part 2	Cardinal Manifestations and Presentation of Diseases
Part 3	Pharmacology
Part 4	Oncology and Hematology
Part 5	Infectious Diseases
Part 6	Disorders of the Cardiovascular System
Part 7	Disorders of the Respiratory System
Part 8	Critical Care Medicine
Part 9	Disorders of the Kidney and Urinary Tract
Part 10	Disorders of the Gastrointestinal System
Part 11	Immune-Mediated, Inflammatory, and Rheumatologic Disorders
Part 12	Endocrinology and Metabolism
Part 13	Neurologic Disorders
Part 14	Poisoning, Drug Overdose, and Envenomation
Part 15	Disorders Associated with Environmental Exposures
Part 16	Genes, the Environment, and Disease
Part 17	Global Medicine
Part 18	Aging
Part 19	Consultative Medicine
Part 20	Frontiers
Part 21	Video Collection
Part 22	Supplementary Topics
Part 23	Atlases
Part 24	Clinical Procedure Tutorials

References

1. Craik EM, Hippocrates. The 'Hippocratic' corpus: content and context. Abingdon, Oxon; New York, NY: Routledge; 2015.
2. Mattern SP. The Prince of Medicine : Galen in the Roman Empire. Oxford: Oxford University Press; 2013.
3. Duncan-Jones RP. The impact of the Antonine plague. Journal of Roman Archaeology. 1996;9:108-36. Epub 2015/02/16.
4. Persaud TVN. A history of anatomy: The post-Vesalian era. Springfield IL: Charles C Thomas Publisher; 1997.
5. Sennert D. De febribus libri IV. Wittebergae: apud Zachariam Schurerum; 1619.
6. Sennert D. Practicae medicinae liber primus-sextus. Wittebergae: Sumtibus viduae et haered. Zachariae Schureri senioris; 1628-1635.
7. Niemeyer F. Lehrbuch der speciellen Pathologie und Therapie, mit besonderer Rücksicht auf Physiologie und pathologische Anatomie. Berlin: Hirschwald; 1858-61.
8. Fracastoro G. De sympathia et antipathia rerum liber unus. De contagione et contagiosis morbis et curatione libri iii. Venetiis: apud heredes Lucaeantonii Juntae Florentini; 1546.
9. Osler W. The principles and practice of medicine. New York, NY: D. Appleton & Company; 1892.
10. Jameson JL FA, Kasper DL, Hauser SL, Longo DL, Loscalzo J (ed). Harrison's principles of internal medicine. 20th ed. New York: McGraw-Hill; 2018.
