# Supplementary material for: The History of Infectious Diseases and Medicine
Source: Pathogens. 2022 Oct 4;11(10):1147. doi: 10.3390/pathogens11101147 (PMC9609744; doi:10.3390/pathogens11101147)
Supplement: Supplementary file 1 [file pathogens-11-01147-s001.zip › Suppl_Microbiology.pdf]

Major infectious diseases in the history of medicine

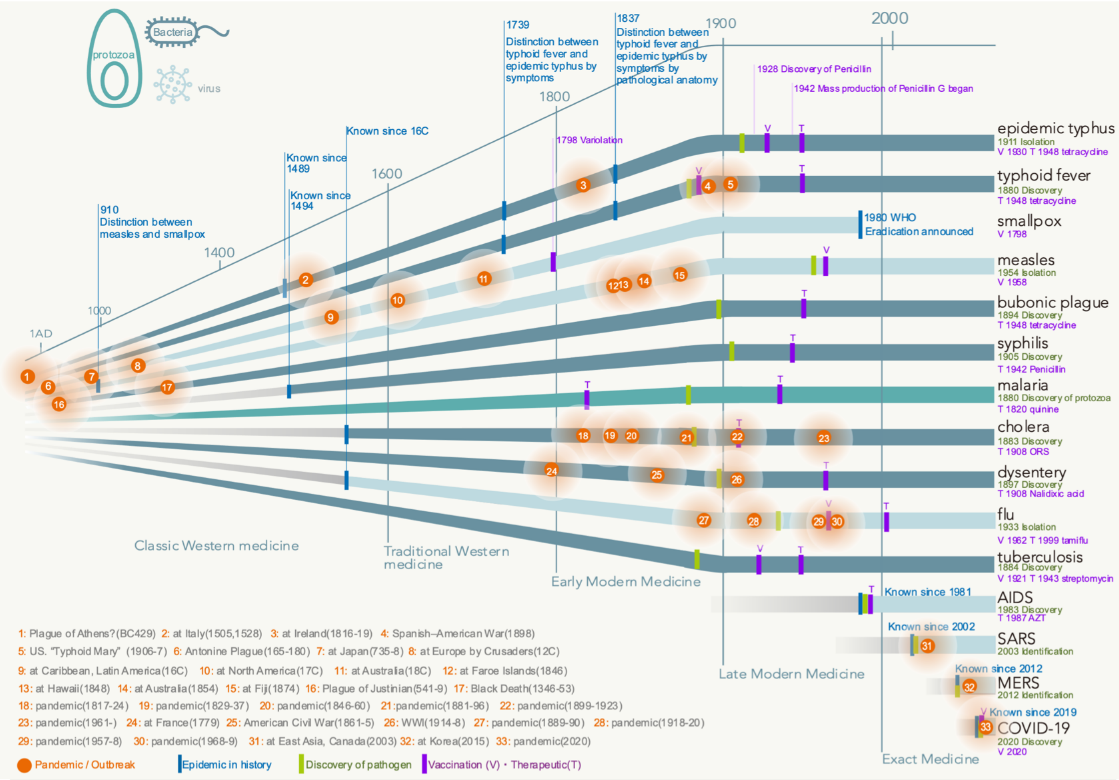

Figure S1. Major infectious disease in the history of medicine.

1. Classic Western medicine (Ancient times to the 15th century)

1.1. The Plague of Athens in the 5th century BC

Ancient Greek culture and medicine were cultivated by the citizens of the polis such as Athens and Sparta. In the second year of the Peloponnesian War (431-404 BC), the city of Athens was devastated by an epidemic called the Plague of Athens (430 BC), killing approximately 25 percent of the population [1, 2]. Thucydides described the epidemic in book two of his *History of the Peloponnesian War*, II.49 as follows[3]:

"*As a rule, however, there was no ostensible cause; but people in good health were all of a sudden attacked by violent heats in the head, and redness and inflammation in the eyes, the inward parts, such as the throat or tongue, becoming bloody and emitting an unnatural and fetid breath. These symptoms were followed by sneezing and hoarseness, after which the pain soon reached the chest, and produced a hard cough. When it fixed in the stomach, it upset it; and discharges of bile of every kind named by physicians ensued, accompanied by very great distress…. Externally the body was not very hot to the touch, nor pale in its appearance, but*

reddish, livid, and breaking out into small pustules and ulcers. But internally it burned so that the patient could not bear to have on him clothing or linen even the of very lightest description; or indeed to be otherwise than stark naked."

The detailed description of the clinical symptoms indicate that the plague must have been an infectious disease, but a definite retrospective diagnosis is not possible. Recent studies have suggested that the plague could have been either a reservoir disease (that is, a zoonotic vector-borne disease) such as typhus or a respiratory disease such as smallpox[1, 2].

1.2. The Antonine Plague in the 2nd century

During the reign of Marcus Aurelius (r. 161-180), the Roman Empire was struck by the Antonine Plague (165-180). The plague was brought to Rome from Seleucia by the Roman army, and spread to the whole empire, causing the estimated deaths of 5-10 million people. A firsthand witness to the plague, Galen testified to the symptoms and described medical histories of the disease, which is today considered smallpox[4]. He wrote in his *Method of Medicine*, V.12[5]:

"At that time, a young man broke out in sores over his whole body on the ninth day, just as did almost all the others who were saved. On that day there was also a slight cough. On the following day, immediately after he washed himself, he coughed more violently and brought up with the cough what they call a scab. And to the person, the sensation was clearly related to the rough artery in the neck near the throat (the trachea), this being the ulcerated part. However, I also opened his mouth and examined the pharynx in case the sore might be somewhere in this…. And in the same way, in those already purged who are going to be saved, exanthemata appear close together over the whole body, in the majority there are sores, while in all there is dryness. And it was quite clear to an observer that, when the blood had been putrefied in those with fever, there was this remnant like ash which nature forced out of the skin, just as with many other superfluities."

Galen described physical symptoms of ulcers in the larynx, trachea, and esophagus as well as exanthema over the whole body, but not the extensive scarring of the skin after recovery which is a characteristic feature of smallpox. The geographical and chronological propagation of the plague has been analyzed on the basis of remaining historical records[4].

1.3. The Plague of Justinian in the 6th century

The Byzantine Empire was stricken by the Plague of Justinian (541-549) during the reign of Justinian I (r. 527-565). The historian Procopius witnessed and described the plague in his *History of the Wars*, II.22. The outbreak started in Egypt (541), spread to the Mediterranean coast (by 544), and remained in Northern Europe and the Arabian Peninsula. He described the symptoms and medical histories as follows[6]:

"*Death came in some cases immediately, in others after many days; and with some the body broke out with black pustules about as large as a lentil and these did not survive even one day, but all succumbed immediately. With many also a vomiting of blood ensued without visible cause and straightway brought death. Moreover I am able to declare this, that the most illustrious physicians predicted that many would die, who unexpectedly escaped entirely from suffering shortly afterwards, and that they declared that many would be saved, who were destined to be carried off almost immediately. So it was that in this disease there was no cause which came within the province of human reasoning; for in all cases the issue tended to be something unaccountable. For example, while some were helped by bathing, others were harmed in no less degree. And of those who received no care many died, but others, contrary to reason, were saved. And again, methods of treatment shewed different results with different patients*."

Procopius's text depicted the fruitless and desperate effort of physicians who could not even determine any effective treatment or reliable prognosis. The plague was most probably a pandemic of bubonic plague[7].

1.4. The Black Death in the 14th century

Medieval Europe was threatened by the Black Death in the mid-14th century. The plague was carried from Crimea to Constantinople by the galleys of Genoese merchants in the summer of 1347, then followed either an eastern path to reach via Egypt to the Middle East, or a western path through the Mediterranean to the whole Western Europe and finally to Russia. The pandemic lasted seven years, from 1346 to 1353, with the epidemic episode in each town continuing for about five to six months on average[8]. (Fig. 3)

The Black Death was a pandemic of bubonic plague that was caused by the *Yersinia pestis* (*Y. pestis*) bacterium. Humans may have been infected by *Y. pestis* via rat fleas as well as by pneumonic transmission. Approximately 50 million people died by the Black Death, which is estimated to be 30 percent to 60 percent of the population of Europe. The medicine practiced in those days possessed neither effective treatment for nor accurate knowledge of the cause of the disease. Traditional regimen (diet, rest, ventilation, phlebotomy), improvement of public health (cleaning roads, limiting activities exposing one to miasmas or "noxious air"), and avoiding contact (lockdown of patients' houses, quarantine, and prohibition of traffic between cities) helped to prevent the spread of the infection to some extent. Wealthy people simply escaped from plague-ridden cities. Boccaccio's *Decameron* (1353) is a literary work constructed around three men and seven women who evacuated from Florence to a secluded villa in the countryside and tell ten stories each to pass the time.

The practice of quarantine, which inspects animals, plants, and food imported from abroad, originated from preventive measures against the Black Death. In 1377, the Republic of Venice enacted a law that forced vessels scheduled for arrival to berth on a small island near a port city of the republic of Ragusa (present-day Dubrovnik, Croatia) for 30 days (later 40 days in Venice) to ensure that there were no infected persons on board. The term "quarantine" was coined from the word for "forty days" (*quarantena*) in the Venetian dialect.

2. Traditional Western medicine in the 16th to 18th centuries

2.1. Syphilis

Syphilis appeared suddenly in Europe at the end of the 15th century. The disease spread rapidly in Italy after the French invasion of Naples in October 1494. People called it the "French disease (*de morbo Gallico*)" and it was greatly feared. It is uncertain whether the disease was imported newly from the New World or had existed long in the Old World in a latent form[9]. Early reports of syphilis attributed a high mortality rate to it, but within a few years it was revealed to be a chronic disease unlike the Black Death. Sufferers were condemned to long lasting pain and disfigured appearance, and were frequently cut off from normal social lives as in the case of leprosy under a similar social stigma. The disease was also called "venereal infection (*lues venerea*)" from the mid-16th century. The Italian physician Girolamo Fracastoro wrote an epic poem titled "*Syphilis sive morbus Gallicus*" ("Syphilis or the French Disease," 1530)[10], although the name of the main character, Syphilis, was not widely used as the name of the disease until the late 18th century.

Syphilis is a sexually transmitted disease caused by the spiral-shaped spirochete *Treponema pallidum* (*T. pallidum*). The clinical course is divided into three stages. In the primary stage, a skin lesion (chancre) develops in the genital area up to three weeks after infection. In the second stage, from the third month after infection, a reddish-pink rash (rose spots) appears throughout the body and eventually disappears. In the tertiary stage, several years later, gummas develop throughout the body, and lesions occur in multiple organs, leading to death (late overt syphilis).

No effective treatment for syphilis has been available until the development of Salvarsan and penicillin in the 20th century. In the 16th century, both guaiacum, gum of *Guaiacum officinalis* from Hispaniola, and a mercury-containing ointment used for skin diseases in Arabic medicine and advocated by Paracelsus, were used widely for the treatment of cutaneous gumma. Controversies raged among physicians for better treatment of syphilis without success.

T. pallidum was first isolated in 1905[11], followed by the development of a serological test (Wassermann reaction) for diagnosis in 1906[12]. An organoarsenic compound Salvarsan was

developed in 1908 by German scientist Paul Erhlich assisted by Japanese bacteriologist Sahachiro Hata, and the antibiotic penicillin, the silver bullet against syphilis, became available in the 1940s.

2.2. Malaria

In ancient times, the recurrent fevers characteristic to the malaria were widespread in the Mediterranean basin. Hippocrates distinguished some types of recurrent fevers in *Epidemics I*[13] and described the tertian fever (section 20) and the quartan fever (section 24) in *Prognostic*[14]. Celsus's *Medicina* (book 3)[15] and Galen's *A Method of Medicine to Glaucon*[16] also described different types of recurrent fevers in addition to other feverish diseases. After the fall of the Roman Empire, Galenic knowledge of feverish diseases was adopted by Avicenna's *Liber canonis* (Liber 4, Fen 1)[17] in Arabia, and to various *Practica* books in Europe such as Gariopontus' *Passionarius* (Liber 6)[18] in the 11th century, Rondelet's *Methodus curandorum omnium morborum corporis humani* (Febribus)[19] in the 16th century, and Sennert's *De febribus* [20] in the early 17th century. In the late 17th century, malaria emerged throughout Europe, as Thomas Sydenham reported the intermittent fever in London in *Methodus curandi febres* (1666)[21] and *Observationes medicae* (1676)[22]. He astutely recommended Peruvian bark (cinchona bark) as remedies for the intermittent fever in *Processus integri* (1693)[23], published after his death.

Malaria is caused by the Plasmodium group of parasites, which is transmitted to humans by the vector of *Anopheles* mosquitoes. The common episode starts with chills, extends through a fever, and ends with sweating, and subsiding fever[1]. Among five *Plasmodium* species that causes infection to human, *Plasmodium falciparum* is the predominant species and the most likely to progress to severe condition with the association of complications[24]. The term "malaria" originates from "bad air" (*mala aria*) in Italian, representing marsh air which was believed to cause intermittent fevers[25]. John MacCulloch wrote a book called *Malaria* (1827)[26], which introduced the term to the English language. The parasites causing it were discovered in the late 19th century[27], and "malaria" became the term to indicate the intermittent fever.

The pharmaceutical compound "quinine" is obtained from the bark of the South American native cinchona tree[27]. The tree bark was brought to Europe in the early 17th century where it was used to treat intermittent fevers[27]. Actual quinine was isolated from cinchona bark in 1820[27]. Moreover, chloroquine, an antimalarial drug with fewer side effects, was synthesized in 1934[27].

Charles Louis Alphonse Laveran discovered the malaria parasite in 1880[28], and Ronald Ross revealed that the disease was transmitted by mosquitoes by locating sporozoites in a mosquito

that had previously fed on an infected patient (1898)[29]. They both received the Nobel Prize in Physiology and Medicine in 1907 and 1902, respectively.

The number of malaria deaths has been diminished by the extermination of *Anopheles* mosquitoes and the dissemination of therapeutic drugs. Nevertheless, as of 2019, malaria still remains a heavy burden in Africa and tropical countries, where it causes an estimated 229 million cases and 409,000 deaths each year[30].

2.3. Smallpox

Although smallpox has been eradicated since 1977, it was once the most severe infectious disease to afflict humankind with a high fatality rate. Onset of the disease is abrupt with a high fever and general pain, followed by a characteristic rash appearing densely on the face, palms, and soles. The rash turns to pustules then forms scabs. In severe cases, massive hemorrhages into the visceral organs could cause to death. The survivors were often seriously scarred extensively on their faces and skin. These characteristics enable the identification of smallpox in the history from the remaining documents with fair certainty.

The Antonine Plague (165) in the Roman Empire is considered to be a smallpox outbreak. In Japan, a smallpox pandemic (735-38, called "*wanzukasa*" at the time) killed four male members of the Fujiwara clan, the most powerful family of imperial regents, having a serious impact on subsequent Japanese history. Smallpox was known to exist all over the world since ancient times, and was often spread through human interaction. During the 12th century, the increased mobility of the Crusaders brought smallpox from the Middle East to Europe. At the end of the 15th century, Columbus's so-called discovery of America brought smallpox to the New World, causing devastating damage to the indigenous people. Sometime after the end of the 18th century, British immigrants brought smallpox to Australia again with equally devastating results.

Smallpox and measles had been confused since ancient times, since both caused skin rashes. The great Persian physician Al Rhazes (Abu Bakr Muhammad ibn Zakariya al-Razi) differentiated smallpox from measles in the 10th century[31]. Avicenna distinguished them in the *Liber canonis*[17], and the knowledge was introduced to Europe in the 12th century.

Smallpox is caused by the *Variola* virus, transmitted to humans by airborne droplets with a mortality rate of 50 percent at worst. Prevention by vaccination is effective, and at first variolation was used. In China, dried smallpox scabs were inoculated into the nostrils. By the mid-17th century, variolation was practiced in southern China, and Emperor Kangxi of the Qing dynasty variolated his children in the 1670s, and the practice became widespread after that time[32]. In western countries, variolation was practiced by folk healers[1]. In 1714 two physicians Emmanuel Timonis and Jacob Pylarini reported in an English journal about the variolation

practices adopted in Turkey and Constantinople[33-35]. Lady Mary Wortley Montagu, the wife of the English ambassador to Turkey, had her son variolated in 1718 while staying at Constantinople and later her daughter in 1721 after returning to England[33]. Variolation was not immediately accepted due to the risk of developing smallpox infection, but it became widespread in Europe after the 1760s.

The English physician-scientist Edward Jenner developed smallpox vaccination using cowpox virus. Jenner's concept of vaccination was based on the observation that milkmaids who were previously exposed to cowpox were immune to smallpox. He vaccinated several people with cowpox and exposed them to smallpox to see if they would develop the illness. When they did not, he published his successful results in 1798[36], followed by wide acceptance of the method in England. His publication was translated into German, French, Spanish, Dutch, Italian, Portuguese, and Latin, leading to the method being introduced all over the world. Strategic immunization by vaccination was later conducted on a worldwide level, under the guidance of the WHO. The last known natural case of smallpox was reported in 1977, and the WHO declared smallpox completely eradicated in 1980[37].

3. Early Modern Medicine (19th century)

3.1. Cholera

Cholera is manifested by acute diarrhea along with severe dehydration and associated physical symptoms. It had long been endemic in the Indian continent and had been recorded by Europeans who visited there in the 16th century. As a consequence of enhanced human mobility in the 19th century, cholera spread worldwide and caused seven pandemics in 1817-24, 1829-37, 1846-60, 1863-75, 1881-96, 1899-1923, and 1961-to date.

The term "cholera" has been used to describe certain illnesses since the time of Hippocrates, and an ancient Roman writer named Celsus also mentioned it in his *De medicina*[15]. Although these are now considered to be caused by different pathogens based on current knowledge of bacterial species, they showed the symptoms of cholera-like sporadic diarrheal diseases, hence "cholera" was used by Thomas Sydenham in his description of a 1669 epidemic in London in his *Observationes medicae*[22].

Cholera is an infectious disease caused by *V. cholerae*, which is transmitted orally through drinking water. *V. cholerae* colonizes the small intestine and produces cholera toxin that activates the G-protein of intestinal epithelial cells, which in turn leads to the increased concentration of intracellular cAMP, resulting in secretion of chloride ions and severe diarrhea. Due to severe dehydration, cholera causes sunken eyes, cyanosis, muscle spasm, decreased blood pressure, and eventually death.

Primary prevention is the decontamination of fecal matter in drinking water. The English physician John Snow traced the source of a cholera outbreak in London by the distribution of the patients and identified the public water pump on Broad Street. He removed the handle of the water pump during the outbreak of cholera in 1854[38]. Robert Koch discovered cholera pathogen through the outbreak investigation in Egypt and India (1884) [39]. Oral rehydration salts (ORS) for the effective treatment of cholera-associated dehydration was advanced by Leonard Rogers and Maxwell Mackelvie in 1908[40]. Cholera toxin was identified by Indian physician and scientist Shambhu Nath De in 1959[41].

3.2. Dysentery

Dysentery is a disease in which lesions occur in the large intestine and causes stools with blood or mucus to be frequently excreted, accompanied by lower abdominal pain, tenesmus, and fever. *Shigella dysenteriae* (*S. dysenteriae*) discovered by Kiyoshi Shiga is known to be the most contagious form of it. Dysentery symptoms are also caused by other *Shigella* species, *Salmonella*, *Campylobacter*, Shiga toxin-producing *Escherichia coli* (STEC), *Yersinia*, and a type of amoeba *Entamoeba histolytica*.

The symptoms of dysentery such as frequent excretion of bloody stools and mucus and abdominal pains have been known since ancient times. The term dysentery [δυσεντερία] appears in *Corpus Hippocraticum* to explain intestinal disease, as well as in chapter 2 in book 6 of Galen's *On the Affected Parts*[42]. Although dysentery is not accounted for in Avicenna's *Liber canonis*[17] and Jean Fernell's *Medicina* (1554) [43], it is mentioned as a gastrointestinal disorder in several books, including Guillaume Rondelet's *Methodus curandorum* (1567) [19] and Daniel Sennert's *Practicae medicinae* (1628-35)[44], and François Boissier de Sauvages de Lacroix classified it as the 10th genus of 2nd order "Alvifluxus" in the class 9 "fluxus" in *Nosologia Methodica* (1763) [45].

S. dysenteriae was discovered by Japanese physician and bacteriologist Kiyoshi Shiga in 1897[46]. Partial purification of Shiga toxin in sufficient quantity for research was obtained in 1953[47]. In 1977, a cytotoxin produced by *Escherichia coli*（*E. coli*）that can kill Vero cells was reported and named verotoxin, which was later found to be the same as the Shiga toxin[48]. Later, foodborne pathogen *E. coli* O157:H7 carrying Shiga toxin caused outbreaks in the US (1982) and Japan (1990) and has had a significant impact on society.

4. Late Modern Medicine (20th century)

4.1. Influenza

Influenza is a viral disease that infects humans, as well as mammals such as livestock and birds, and is spread through respiratory droplets. Onset is acute, followed by respiratory symptoms such as runny nose, cough, and sore throat, but also systemic symptoms such as fever, headache, myalgia, and malaise are common. Among four types of influenza viruses, type A and B viruses circulate among humans, and type A viruses occasionally acquire mutations and cause a pandemic[49]. Avian or swine influenza viruses can infect humans and are thus a matter of concern. By the mid-19th century, the records of six pandemics remain (1510?, 1557?, 1580, 1729, 1781-82, and 1830-33)[1, 50], and detailed records have been kept since the late 19th century with increased awareness of public health.

1. Russian flu (1889-90): Initially reported in the Russian Empire city of Bukhara (present-day Uzbekistan) in May 1889, it spread to Europe via rail transport and the US via maritime transport. The pandemic killed an estimated one million people by 1890.
2. Spanish flu (1918-20): The first wave (March 1918) began with the case of the US army and spread to Europe, Africa, Asia, and Australia by July. The second wave (August 1918) began from the US, France, and West Africa, spread throughout the world, and lasted until the end of the year 1918. The third wave (January 1919) began from Australia, spread to Europe and the US, and lasted until summer 1919. The outbreak then spread to the Southern Hemisphere and Japan and lasted until the spring of 1920. Eventually, 500 million people were infected worldwide and an estimated 100 million died.
3. Asian flu (1957-58): While the outbreak began in southwestern China (February 1957), the global pandemic began in Hong Kong (April 1957) and spread rapidly to Asia, Australia, the US, and Europe by airways. Eventually, 500 million people were infected worldwide and an estimated 1 million died.
4. Hong Kong flu (1968-69): A sudden outbreak began in Hong Kong (June-July, 1968), with the first wave spreading to Asia, Europe, and the US, and a bigger second wave in the following year. Since then, it has been circulating globally as seasonal flu.

Hand-washing hygiene and droplet-contact prevention are essential components to prevent the transmission of pathogens[51]. Neuraminidase inhibitors have been developed as antiviral drugs. Oral Tamiflu, inhaler Relenza, and Inavir, and intravenous Rapivab are currently available in many countries.

4.2. Tuberculosis

Tuberculosis (TB) is a chronic infection caused by *M. tuberculosis*. It is mainly a lung disease but can also affect other organs throughout the body with various types of disease. Many names have been given it since ancient times, including phthisis (pulmonary tuberculosis), scrofula (cervical lymph node tuberculosis), lupus vulgaris (cutaneous tuberculosis), and miliary tuberculosis (acute tuberculosis).

TB has been known to be around since prehistoric times by tracing of lesions left in mummies. In ancient Greece, the words "*phthisis* [φθίσις]" appear in the Hippocratic corpus and Galenic corpus, however, not all of them may have been pulmonary TB. Lung ulcer and labor cough as a type of pulmonary disease are described in Avicenna's *The Canon of Medicine*, Rondelet's medical practice book, and Sennert's *Practicae medicinae*, thus tuberculosis was recognized as a pulmonary disease. Moreover, Avicenna and Sennert mentioned *scrofula* (disorder on the cervical or body surface). German physician Johann Lukas Schönlein proposed a concept of TB by assembling various types of diseases. He introduced TB in chapter XII "Tuberkeln" and chapter XII "Phthisen" in his book *Allgemeine und spezielle Pathologie und Therapie* (General and special pathology and therapy, 1834). With the Industrial Revolution and urbanization in the 19th century, TB spread in Western countries, causing about 40% of working-class deaths in cities.

Koch discovered the tuberculosis bacterium in 1882[52]. He extracted tuberculin, bacterial proteins with glycerin, which had no therapeutic effect as expected but was later used for the diagnosis of TB infection. Bacille Calmette-Guérin (BCG), made by the attenuation of bovine TB in 1921, is used as a vaccine. Radiography using X-rays, which was discovered by Wilhelm Conrad Röntgen, enabled the diagnosis of pulmonary diseases including TB, and fluoroscopy was first introduced in 1936 is widely used in medical checkups today.

There had long been no effective cure for TB, and it was feared as a deadly disease. Treatment of TB was limited to a nourishing and resting regimen, and the patients were admitted to a TB sanatorium. Artificial pneumothorax[53], thoracoplasty[54], and lung resection[55] were also used as surgical treatments. Radiotherapy was also performed and the best results were obtained when applied early in ascitic forms of peritoneal tuberculosis, as well as tuberculosis mucosal lesions and lymphadenitis[56, 57]. The introduction of streptomycin (1943), the first antibiotic active against TB, was the game-changer in the treatment of TB. Together with the improvement of public health and nutrition, the number of deaths caused by TB has dropped dramatically after WWII.

5. Exact Medicine (1990 - to date)

5.1. Antimicrobial resistance (AMR)

The golden age of new antibiotic discoveries ended in the mid-20th century[58]. Bacteria always find a way to develop resistance to newly-introduced antibiotics, leading to a vicious cycle. Of the 14 antibiotics approved between 2014 to 2020, only one emerged in a novel class (pleuromutilin), and two emerged in novel β-lactamase inhibitor combinations[59]. In 2014, an AMR review that was commissioned by the UK government, the so-called O'Neill Report, estimated that without finding proactive strategy to control the emergence of drug resistance, 10 million lives per year and $100 trillion USD of economic output are at risk due to AMR by 2050[60]. The Global Action Plan on Antimicrobial Resistance (GAP) was released in 2015, and the One Health approach that aims to achieve optimal health outcomes, recognizing the interconnection between humans, animals, plants, and their shared environment, is widely promoted by the WHO, the Food and Agriculture Organization of the United Nations (FAO), and the World Organisation for Animal Health (OIE)[61, 62]. In 2015, WHO launched the Global Antimicrobial Resistance and Use Surveillance System (GLASS), which was the first globally standardized approach on surveillance and research of AMR[63]. Without effective global action, treatments of infectious disease may revert to the level of previous periods without antimicrobial drugs.

As early as 1945, Fleming warned about the possible scenario of the emergence of penicillin-resistant strains selected by the insufficient concentrations of the antibiotic in his Nobel lecture[64]. Despite Fleming's warning, sublethal concentrations of antibiotics fed to livestock became common practice in the US since 1949 and UK since 1953, expecting to promote better livestock growth[65]. The report of the Joint Committee on the Use of Antibiotics in Animal Husbandry and Veterinary Medicine in 1969, also known as the Swann Report, was the first to alert the world to the emergence of antibiotics-resistant strains caused by overuse of antibiotics in animals[65]. Still today, livestock consumes an estimated 50-80% of all antibiotics consumed for therapeutic, disease prevention, and growth promotion purposes in many developed countries[66].

Although the current circumstances of the AMR threat are the result of anthropogenic activities that provide constant selective pressure for resistant strains[67], antimicrobial resistance genes have existed in the terrestrial ecosystems before the discovery of antibiotics[68]. In fact, some antimicrobial resistance genes are known to exist long before human history. Among them, the origin of the *mecA* gene has been well investigated. The *mecA* gene that encodes the penicillin-binding protein 2' (PBP2') with a reduced affinity of β-lactam antibiotics is carried by

a mobile genetic element, staphylococcal cassette chromosome *mec* (SCC*mec*). Acquisition of SCC*mec* into methicillin-susceptible *S. aureus* (MSSA) chromosome generates the methicillin-resistant *S. aureus* (MRSA). The origin of the *mecA* gene was discovered on the chromosome of *S. fluerettii*, one of the oldest *Staphylococcus* species[69]. The *mecA* gene of *S. fluerettii* locates next to the genes constituting an important metabolic pathway and has no association with SCC*mec*[69]. The corresponding gene loci were also found in the chromosome of two species sharing a common ancestor, *S. sciuri* and *S. vitulinus*, suggesting acquisition of the *mecA* gene is not a recent event. Carrying a *mecA* gene might be an advantage for the ancestral staphylococci to survive in an environment surrounded by fungi and *Actinobacteria* that produce β-lactam antibiotics. However, the resistance gene was truncated or deleted from the chromosomes of the descendants of the major staphylococcal species, including *S. aureus*[70]. Colonization to mammals might have involved the deletion of *mecA* since bacteria were no longer exposed to environmental β-lactams[70]. The *mec* gene had existed in the environmental reservoir for millions of years until clinical application of the β-lactams. Localization of the *mec* on the MRSA chromosome was discovered in 1975[71].

The concept of the antibiotic resistome was first introduced by D'Costa VM et al. in 2006[68]. A number of studies focusing on resistome have indicated that AMR is ancient, ubiquitous, and circulating among humans, animals, and environment[72]. Similar to the historical events of infectious diseases we focus on in this study, anthropogenic activities play significant roles in the emergence and selection of AMR, which is considered a global pandemic in the era of exact medicine.

5.2. AIDS

AIDS (acquired immunodeficiency syndrome) is caused by infection with human immunodeficiency virus (HIV) that is transmitted by contaminated blood or body fluid. HIV is believed to have originated in primates that inhabited West and central Africa. The first HIV case was recognized in the US in 1981, with subsequent cases being reported one after another from, among others, members of the gay community, patients who received blood transfusions, and drug addicts. In 1983, HIV was isolated by French virologists Luc Montagnier and Françoise Barré-Sinoussi who were awarded the 2008 Nobel Prize for Physiology and Medicine[1]. Following the approval of AZT (1987), several anti-HIV drugs became available, which meant that the treatment of HIV progressed dramatically. AIDS, once a deadly disease, has now been shifted to a chronic one that is treated outpatient. Worldwide (in 2020), there were an estimated 1.5 million new cases and 690,000 deaths, with 77.5 million cumulative cases and 34.7 million deaths.

5.3. COVID-19

COVID-19 is an infectious disease caused by a novel coronavirus (SARS-CoV-2: Severe acute respiratory syndrome coronavirus 2). The outbreak began at the end of 2019, and on March 11, 2020, WHO declared a global pandemic[73]. Since then, COVID-19 has continued to have a massive impact on global society, economy, medicine, lifestyle, and mental health.

The pathophysiological mechanisms of the novel virus are being actively studied. After a three to five day period of mean incubation, the common symptoms patients develop include fever, respiratory distress, headache, and fatigue. Some patients develop severe symptoms of widespread pneumonia with characteristic ground-glass like CT-image and respiratory distress. For severe cases, oxygen support, mechanical ventilation, or extracorporeal membrane oxygenation (ECMO) are needed to address respiratory failure, the major risk of death. The disease is mainly transmitted by contaminated droplets, but also infection by aerosols indoors or through contact transmission have been known, therefore, the use of face masks and avoiding the so-called Three Cs (closed spaces, crowded places, and close-contact settings) are effective for prevention. Vaccines have been developed rapidly and several have been allowed for emergency use.

References

1. Kiple KF. The Cambridge historical dictionary of disease. Cambridge MA: Cambridge University Press; 2012.
2. Littman RJ. The plague of Athens: Epidemiology and paleopathology. Mt Sinai J Med. 2009 Oct;76(5):456-67. PubMed PMID: 19787658. Epub 2009/09/30. eng.
3. Thucydides, Crawley R. The history of the Peloponnesian War. Chicago IL: Henry Regnery; 1974.
4. Duncan-Jones RP. The impact of the Antonine plague. Journal of Roman Archaeology. 1996;9:108-36. Epub 2015/02/16.
5. Galen, Johnston I, Horsley GHR. Method of Medicine, Volume II: Books 5-9. Cambridge MA: Harvard University Press; 2011.
6. Procopius, Downey G, Dewing HB. Procopius. Cambridge MA: Harvard University Press; 1971.
7. Retief F, Cilliers L. The epidemic of Justinian (AD 542): A prelude to the Middle Ages. Acta Theologica. 2010 03/10;26 Supplmentum 7:115-27.
8. Benedictow OJ. The Black Death, 1346-1353: The Complete History. Woodbridge, UK: Boydell Press; 2004.
9. French R, Arrizabalaga J. Coping with the French Disease: University Practitioners' Strategies and Tactics in the Transition from the Fifteenth to the Sixteenth Century. In: French R, Arrizabalaga J, Cunningham A, García-Ballester L, editors.: Medicine from the black death to the French disease. London: Ashgate; 1998. p. 248-87.
10. Fracastoro G. Syphilis, sive morbus gallicus. Veronae: Stefano dei Nicolini de Sabbio e Fratelli; 1530.
11. Schaudinn F, Hoffmann E. Ueber Spirochaetenbefunde im Lymphdrüsensaft Syphilitischer. Dtsch Med Wochenschr. 1905;31(18):711-4.
12. Wassermann A, Bruck C. Experimentelle Studien über die Wirkung von Tuberkelbacillen-Präparaten auf den tuberculös erkrankten Organismus. Dtsch Med Wochenschr. 1906;32(12):449-54.
13. Hippocrates, Jones WHS. Hippocrates, volume I. Cambridge, MA: Harvard University Press; 1923.
14. Hippocrates, Jones WHS. Hippocrates, volume II. Cambridge, MA: Harvard University Press; 1923.
15. Celsus, Spencer WG. Celsus: De Medicina, volume I-III. Cambridge, MA: Harvard University Press; 1971.

16. Galen, Johnston I. On the Constitution of the Art of Medicine. The Art of Medicine. A Method of Medicine to Glaucon. Cambridge, MA: Harvard University Press; 2016.
17. Avicenna. Liber Canonis, De medicinis cordialibus, et Cantica. Basileae: per Joannes Hervagios; 1556.
18. Gariopontus. Passionarius Galeni ... egritudines a capite ad pedes usque complectens. Lugduni: In edibus Antonii Blanchardi, sumptu Bartholomei Trot; 1526.
19. Rondelet G. Methodus curandorum omnium morborum corporis humani ... Ejusdem De dignoscendis morbis. De febribus. De morbo Gallico. de [medicamentis] internis & externis. De pharmacopolarum officina. De fucis. Parisiis: apud Jacobum Maceum; 1567.
20. Sennert D. De febribus libri IV. Wittebergae: apud Zachariam Schurerum; 1619.
21. Sydenham T. Methodus curandi febres, propriis observationibus superstructa. Londini: Impensis J. Crook; 1666.
22. Sydenham T. Observationes medicae circa morborum acutorum historiam et curationem. Londini: Typis A. C. impensis Gualteri Kettilby; 1676.
23. Sydenham T. Processus integri in morbis ferè omnibus curandis. Londini: Impensis Sam. Smith & Benj. Walford & J. Knapton; 1693.
24. Malaria-Parasite Biology. Centers for DiseaseControl and Prevention (CDC). Available from: https://www.cdc.gov/dpdx/malaria/index.html [Accessed 2021-09-24].
25. Hempelmann E, Krafts K. Bad air, amulets and mosquitoes: 2,000 years of changing perspectives on malaria. Malar J. 2013 Jul 9;12:232. PubMed PMID: 23835014. Pubmed Central PMCID: PMC3723432. Epub 2013/07/10. eng.
26. Macculloch J. Malaria: an essay on the production and propagation of this poison. London: Longman, Rees, Orme, Brown, and Green; 1827.
27. Arrow K, Panosian C, Gelband H, (eds). Saving lives, buying time economics of malaria drugs in an age of resistance. Washington (DC): National Academies Press; 2004.
28. Laveran CLA. Nature parasitaire des accidents de l'impaludisme, description d'un nouveau parasite trouvé dans le sang des malades atteints de fièvre palustre. Paris: Baillière; 1881.
29. Ross RIMS. Report on the Cultivation of Proteosoma, Labbé, in Grey Mosquitos. Ind Med Gaz. 1898 Nov;33(11):401-8. PubMed PMID: 29001935. Pubmed Central PMCID: PMC5141207. Epub 1898/11/01. eng.
30. Malaria. World Health Organization (WHO) Available from: https://www.who.int/news-room/fact-sheets/detail/malaria.
31. Rhazes. De variolis et morbillis, arabice et latine: cum aliis nonnullis eiusdem argumenti / cura et impensis Iohannis Channing. Londini: excudebat Guilielmus Bowyer; 1766.

32. Fukushi Y. Historical Development of Preventive Inoculation in China : Focusing on the Vaccination Policy [in Japanese]. Kaigai Shakaihosho Kenkyu. 2015;192:33-45.

33. Eriksen A. Smallpox inoculation: translation, transference and transformation. Palgrave Communications. 2020 2020/03/24;6(1):52.

34. Timonius EWJ. V. An account, or history, of the procuring the smallpox by incision, or inoculation; as it has for some time been practised at Constantinople. Philosophical Transactions of the Royal Society of London. 1714;29(339):72-82. No Linguistic Content.

35. Pylarini G. Nova et tuta variolas excitandi per transplantationem methodus, nuper inventa & in usum tracta. Philosophical Transactions of the Royal Society of London. 1714;29(347):393-9.

36. Jenner E. An inquiry into the causes and effects of the variolae vaccinae, a disease discovered in some of the western counties of England, particularly Gloucestershire, and known by the name of the cow pox. London: Printed for the author; 1798.

37. Smallpox. World Health Organization (WHO) [Accessed 2021-09-25]. Available from: https://www.who.int/health-topics/smallpox.

38. Snow J. Report on the cholera outbreak in the Parish of St. James, Westminster, during the autumn of 1854. Presented to the vestry by the Cholera Inquiry Committee, July 1855. London: Churchill; July 1855.

39. Koch R. Ueber die Cholerabakterien. Dtsch Med Wschr. 1884;10:725-8.

40. Rogers LIMS, Mackelvie M. Note on the value of large quantities of hypertonic salt solutions in transfusion for cholera. Ind Med Gaz. 1908 May;43(5):165-7. PubMed PMID: 29006556. Pubmed Central PMCID: PMC5183079. Epub 1908/05/01. eng.

41. De SN. Enterotoxicity of bacteria-free culture-filtrate of *Vibrio cholerae*. Nature. 1959 May 30;183(4674):1533-4. PubMed PMID: 13666809. Epub 1959/05/30. eng.

42. Galen, Siegel RE. Galen on the affected parts : translation from the Greek text with explanatory notes. Basel, New York: Karger; 1976.

43. Fernel JF. Medicina. Lutetiae Parisiorum: apud Andream Wechelum; 1554.

44. Sennert D. Practicae medicinae liber primus-sextus. Wittebergae: Sumtibus viduae et haered. Zachariae Schureri senioris; 1628-1635.

45. Sauvages F. Nosologia methodica, sistens morborum classes, genera et species juxta Sydenhami mentem et botanicorum ordinem. Amstelodami: sumptibus fratrum de Tournes; 1763.

46. Shiga K. [Dysentery pathogen research report (First)] Saikingaku zasshi. 1897;1897(25):790-810.

47. Van Heyningen WE, Gladstone GP. The neurotoxin of Shigella shigae. I. Production, purification and properties of the toxin. Br J Exp Pathol. 1953 Apr;34(2):202-16. PubMed PMID: 13051531. Pubmed Central PMCID: PMC2073501. Epub 1953/04/01. eng.
48. Kaper JB, O'Brien AD. Overview and historical perspectives. Microbiol Spectr. 2014 Dec;2(6). PubMed PMID: 25590020. Pubmed Central PMCID: PMC4290666. Epub 2015/01/16. eng.
49. World Health Organization. Influenza (Seasonal). Available from: https://www.who.int/news-room/fact-sheets/detail/influenza-(seasonal) [Accessed 2021-10-07].
50. Potter CW. A history of influenza. J Appl Microbiol. 2001 Oct;91(4):572-9. PubMed PMID: 11576290. Epub 2001/09/29. eng.
51. World Health Organization. Infection prevention and control during health care for confirmed, probable, or suspected cases of pandemic (H1N1) 2009 virus infection and influenza-like illnesses Available from: https://www.who.int/csr/resources/publications/cp150_2009_1612_ipc_interim_guidance_h1n1.pdf [Accessed 2021-10-07].
52. Koch R. Die ƒtiologie der Tuberkulose (1882). 2018:113-31. No Linguistic Content.
53. Forlanini C. A contribuzione della terapia chirurgica nella tisi del polmone. Ablazione del polmone? Pneumotorace artificiale? Scritti di Carlo Forlanini; L Cappelli Editore. Roma, Italy1928. p. 400-32.
54. Overholt RH. Selective thoracoplasty in the treatment of pulmonary tuberculosis. Ann Surg. 1935 Jan;101(1):576-93. PubMed PMID: 17856487. Pubmed Central PMCID: PMC1391095. Epub 1935/01/01. eng.
55. Brown CJ. Resection of lung tissue for pulmonary tuberculosis. Med J Aust. 1949 Feb 12;1(7):185-9. PubMed PMID: 18114044. Epub 1949/02/12. eng.
56. Mayer E, Dworkin M. Roentgen and Light Therapy of Intestinal and Peritoneal Tuberculosis. Radiology. 1938;31(1):35-41.
57. Samson PC, Barnwell J, Littig J, Bugher JC. Tuberculous tracheobronchitis. Journal of the American Medical Association. 1937;108(22):1850-5.
58. Lobanovska M, Pilla G. Penicillin's Discovery and Antibiotic Resistance: Lessons for the Future? Yale J Biol Med. 2017;90(1):135-45. PubMed PMID: 28356901. eng.
59. The Pew Charitable Trust. “Antibiotics Currently in Global Clinical Development” 2014 (Updated 2021). Available from: https://www.pewtrusts.org/en/research-and-analysis/data-visualizations/2014/antibiotics-currently-in-clinical-development.
60. O’Neill J. Tackling drug-resistant infections globally: final report and recommendations. 2016.

61. World Health Organization "Antimicrobial resistance". 2021. Available from: https://www.who.int/news-room/fact-sheets/detail/antimicrobial-resistance.
62. Centers for Disease Control and Prevention "One Health Basics" 2018. Available from: https://www.cdc.gov/onehealth/basics/index.html.
63. World Health Organization. Global Antimicrobial Resistance and Use Surveillance System (GLASS). Available from: https://www.who.int/initiatives/glass.
64. Fleming A. Penicillin (Nobel Lecture) 1945. Available from: https://www.nobelprize.org/uploads/2018/06/fleming-lecture.pdf.
65. Swann M. Report / Joint Committee on the use of Antibiotics in Animal Husbandry and Veterinary Medicine. 1969.
66. Cully M. Public health: The politics of antibiotics. Nature. 2014 May 1;509(7498):S16-7. PubMed PMID: 24784425. Epub 2014/05/03. eng.
67. Davies J, Davies D. Origins and evolution of antibiotic resistance. Microbiol Mol Biol Rev. 2010 Sep;74(3):417-33. PubMed PMID: 20805405. Pubmed Central PMCID: PMC2937522. Epub 2010/09/02. eng.
68. D'Costa VM, McGrann KM, Hughes DW, Wright GD. Sampling the antibiotic resistome. Science. 2006 Jan 20;311(5759):374-7. PubMed PMID: 16424339. Epub 2006/01/21. eng.
69. Tsubakishita S, Kuwahara-Arai K, Sasaki T, Hiramatsu K. Origin and molecular evolution of the determinant of methicillin resistance in staphylococci. Antimicrob Agents Chemother. 2010 Oct;54(10):4352-9. PubMed PMID: 20679504. Pubmed Central PMCID: PMC2944575. Epub 2010/08/04. eng.
70. Hiramatsu K, Ito T, Tsubakishita S, Sasaki T, Takeuchi F, Morimoto Y, et al. Genomic Basis for Methicillin Resistance in *Staphylococcus aureus*. Infect Chemother. 2013 Jun;45(2):117-36. PubMed PMID: 24265961. Pubmed Central PMCID: PMC3780952. Epub 2013/11/23. eng.
71. Sjöström JE, Löfdahl S, Philipson L. Transformation reveals a chromosomal locus of the gene(s) for methicillin resistance in *Staphylococcus aureus*. J Bacteriol. 1975 Sep;123(3):905-15. PubMed PMID: 125746. Pubmed Central PMCID: PMC235813. Epub 1975/09/01. eng.
72. Kim DW, Cha CJ. Antibiotic resistome from the One-Health perspective: understanding and controlling antimicrobial resistance transmission. Exp Mol Med. 2021 Mar;53(3):301-9. PubMed PMID: 33642573. Pubmed Central PMCID: PMC8080597. Epub 2021/03/02. eng.
73. Cucinotta D, Vanelli M. WHO Declares COVID-19 a Pandemic. Acta Biomed. 2020 Mar 19;91(1):157-60. PubMed PMID: 32191675. Pubmed Central PMCID: PMC7569573 stock

ownership, equity interest, patent/licensing arrangement etc.) that might pose a conflict of interest in connection with the submitted article. Epub 2020/03/20. eng.
74. COVID19 Vaccine tracker. Available from: https://covid19.trackvaccines.org/.
75. Fleischmann RD, Adams MD, White O, Clayton RA, Kirkness EF, Kerlavage AR, et al. Whole-genome random sequencing and assembly of *Haemophilus influenzae* Rd. Science. 1995 Jul 28;269(5223):496-512. PubMed PMID: 7542800. Epub 1995/07/28. eng.
76. U.S. National Library of Medicine. Genome Information by Organism. Available from: https://www.ncbi.nlm.nih.gov/genome/browse#!/prokaryotes/.
77. NIH News Release. NHGRI Seeks Next Generation of Sequencing Technologies. Available from: https://www.genome.gov/12513210/2004-release-nhgri-seeks-next-generation-of-sequencing-technologies.
78. Mardis ER. A decade's perspective on DNA sequencing technology. Nature. 2011 Feb 10;470(7333):198-203. PubMed PMID: 21307932. Epub 2011/02/11. eng.
79. Kuroda M, Ohta T, Uchiyama I, Baba T, Yuzawa H, Kobayashi I, et al. Whole genome sequencing of meticillin-resistant *Staphylococcus aureus*. Lancet. 2001 Apr 21;357(9264):1225-40. PubMed PMID: 11418146. Epub 2001/06/22. eng.
80. World Health Organization. GLASS whole-genome sequencing for surveillance of antimicrobial resistance.2020.
81. Majander K, Pfrengle S, Kocher A, Neukamm J, du Plessis L, Pla-Díaz M, et al. Ancient bacterial genomes reveal a high diversity of Treponema pallidum strains in early modern Europe. Curr Biol. 2020 Oct 5;30(19):3788-803.e10. PubMed PMID: 32795443. Epub 2020/08/17. eng.
